# Pediatric Cushing's Disease and Pituitary Incidentaloma: Is This a Real Challenge?

**DOI:** 10.1155/2014/851942

**Published:** 2014-10-20

**Authors:** Rosa Maria Paragliola, Pietro Locantore, Alfredo Pontecorvi, Salvatore Maria Corsello

**Affiliations:** Endocrinology Unit, Catholic University School of Medicine, Largo Gemelli No. 8, 00168 Rome, Italy

## Abstract

Cushing's disease (CD) is the most common cause of endogenous Cushing's syndrome in children and adolescents and represents a rare cause of short stature. A 14-year-old boy came to our attention for progressive weight gain and short stature. At examination, height was 140 cm (3rd centile) and weight was 37.7 kg (10th centile). Tanner stage was G2, PH 3, testis 3 mL. Hypothyroidism and growth hormone deficiency were excluded. A marked increase of urinary free cortisol, a nonsuppressible serum cortisol after Liddle 1 test, and an elevated ACTH value confirmed the diagnosis of ACTH dependent Cushing's syndrome. Pituitary magnetic resonance imaging (MRI) showed a left microadenoma and a right focal area of lesser enhancement. Therefore, bilateral inferior petrosal sinus sampling (BIPSS) with CRH stimulation was performed to obtain an accurate preoperative localization of the adenoma: the interpetrosal sinus ACTH gradient indicated lateralization of ACTH secretion to the left side. The patient underwent transsphenoidal surgery with selective microadenomectomy, with an immediate ACTH decline in the postoperative phase. Histology confirmed the diagnosis of corticotrophic pituitary adenoma. Glucocorticoid replacement therapy was instituted. Clinical examination demonstrated a rapid catch-up growth (10th centile), with a normalization of body mass index and an adequate pubertal development.

## 1. Introduction

Cushing's disease (CD) is the most common cause of endogenous hypercortisolism in children and adolescents and represents a rare cause of short stature. Growth failure and weight gain are frequently observed in subjects with ACTH-dependent hypercortisolism, while in ACTH-independent Cushing's syndrome the secretion of adrenal androgens may lead to an acceleration of bone age and eventually compromise growth potential [1, 2]. The most important factor causing growth failure in pediatric CD is the prolonged overexposure to glucocorticoids, although the decrease in free IGF1 and the resistance to IGF1 and other growth factors can also contribute; moreover, concomitant GH deficiency, both before and after surgery, has been described [3]. Early diagnosis and adequate treatment are important prerequisites to assure a catch-up growth after remission, avoiding compromising the final adult height: however, diagnosis and treatment of pediatric CD often represent a challenge for the clinicians.

## 2. Case Report

A 14-year-old boy came to our attention for progressive weight gain and short stature. Family history was negative for endocrine diseases, and his final estimated target height was 175 cm (~50th centile).

Birth length (53 cm) and weight (3100 gr) were normal and clinical history was negative for use of glucocorticoids. Patient had regularly played soccer up to 5-6 months before, but he had to stop because of the onset of severe fatigue.

At physical examination, height was 140 cm (3rd centile) and weight was 37.7 kg (10th centile). His weight increased by 5 kg in the last three months. Blood pressure was normal and there were not striae rubrae, acanthosis, facial plethora, hirsutism, and dorsocervical or supraclavicular fat pad. Tanner stage was G2, PH 3, testes 3 mL. Bone age was consistent with chronological age. Routine blood text exams did not show abnormalities and in particular hypokaliemia was not detected. Hypothyroidism and growth hormone deficiency were excluded. There was a marked increase of urinary free cortisol, with a nonsuppressible serum cortisol after low-dose dexamethasone suppression test and detectable ACTH value which confirmed the diagnosis of ACTH-dependent Cushing's syndrome. Hormonal laboratory evaluations are as follows:

 FT_3_: 3.07 pmol/L (n.v. 3.53–6.45),
 FT_4_: 12.98 pmol/L (n.v. 11.03–20.12),
 TSH: 0.66 *µ*U/mL (n.v. 0.35–2.8),
 FSH: 0.5 mU/mL,
 LH: 0.1 mU/mL,
 testosterone: 0.92 nmol/L,
 IGF_1_: 74.67 nmol/L (n.v. for age 42–103),
 serum cortisol (morning): 872 nmol/L (n.v. 221–607),
 ACTH: 21.77 pmol/L (n.v. 2.22–12.22),
 urinary free cortisol: 173 *µ*g/die (n.v. 36.5–137),
 serum cortisol after low dose dexamethasone suppression test (dexamethasone 0.5 mg every 6 hours for 48 hours): 317.28 nmol/L,
 serum cortisol after high dose dexamethasone suppression test (dexamethasone 2 mg every 6 hours for 48 hours): 87.18 nmol/L

(n.v.: normal values).

The MRI of the pituitary gland showed a left microadenoma (9 mm) and another right focal area of lesser enhancement of about 5-6 mm. Therefore, bilateral inferior petrosal sinus sampling (BIPSS) with CRH stimulation was performed to obtain an accurate preoperative localization of the adenoma: the interpetrosal sinus ACTH gradient indicated lateralization of ACTH secretion to the left side (Table 1).

The patient underwent transsphenoidal surgery with selective microadenomectomy, followed by an immediate ACTH and serum cortisol decline (<1.1 pmol/L and 28 nmol/L, resp.) in the postoperative phase. Histology confirmed the diagnosis of “corticotrophic left pituitary microadenoma.” Glucocorticoid replacement therapy was instituted and progressively reduced over a 12–14-month period and eventually discontinued. Clinical examination showed a rapid catch-up growth (10th centile), with a normalization of body mass index and an adequate pubertal development (G5, P5, testes 15 mL). Biochemical evaluation did not show any deficiency of pituitary function and, after 5 years of follow-up, the patient can be considered free of disease. At the age of 19 height is 174 cm (~50th centile) according to patient's estimated target height.

## 3. Discussion

Pediatric Cushing's syndrome (CS), caused by prolonged exposure to excessive exogenous or endogenous glucocorticoids, is a rare condition in childhood and adolescence. The incidence of CS in the general population is about 2–5 cases per million per years, and only in 10% of cases this condition is diagnosed in children or adolescents [4].

The most frequent cause of CS is the iatrogenic administration of supraphysiological doses of glucocorticoids, for example, for the treatment of pulmonary, dermatologic, autoimmune, and neoplastic diseases.

Similar to what is observed in adults [5], the most frequent cause of endogenous CS in children and adolescents is Cushing's disease, which consists in ACTH overproduction from a pituitary adenoma, and represents approximately 75% of all cases of CS in children over 7 years. This condition shows a higher prevalence in males than in females in prepubertal age. Under 7 years, adrenal causes of CS are most frequent (adenoma, carcinoma, bilateral hyperplasia, and McCune Albright syndrome). Ectopic ACTH syndrome or CRH-dependent CS is very rare in children and adolescents [5].

The most common presentation of CS in children is growth retardation and weight increases [6] although patients with virilizing adrenal tumors can present with a growth acceleration induced by androgens [7]. Thus, CS should always be considered in the evaluation of short stature in children. Pubertal disorders may be encountered: younger children may present virilization with pseudoprecocious puberty while older children and adolescents may have delayed puberty as a consequence of glucocorticoid-induced hypogonadism [1]. Bone age in children and adolescent with CS is consistent with the chronological age in 81% of cases, while it is accelerated in 8% and delayed in 11%, correlating with early and delayed sexual development, respectively [1]. Other signs such as moon face, acne, hirsutism, hypertension, and striae rubrae may be seen in about half of patients with inappropriate cortisol overproduction [8]. Moreover, hypercortisolism can cause alterations in body composition with increase in visceral adiposity, increased fasting insulin, and decreased bone mass [9]. An increased risk of infections related to immunosuppression can occur.

The most important clinical feature seen in our patient, as well as growth failure and weight gain, was fatigue that had a sudden onset and led to the impossibility to play sport. However, muscle weakness was not present at physical examination: in fact this sign, which has high specificity in adult patients, may be less common in pediatric and adolescent patients [1]. The investigation protocols used for pediatric CS are the same used in adult practice. An appropriate therapeutic approach depends on an accurate diagnosis of the disease. Detailed medical history and clinical evaluation, including review of growth data, are important to make the initial diagnosis of CS, even if variable clinical presentation with subtle signs and symptoms can make the diagnosis difficult. Moreover, rare cases of human glucocorticoid receptor gene mutation have been described both in germline and somatic state in CD. In these cases, a partial glucocorticoid resistance can lead to the relative absence of characteristic signs and symptoms of cortisol overproduction [10]. Briassoulis et al. recently described a pediatric patient with a pituitary ACTH secreting adenoma confirmed by histology, with little clinical evidence of CD. However, in this case, no mutation in glucocorticoid receptor gene was detected [11].

Since the majority of these pediatric patients come to medical evaluation for height failure, other causes of short stature (such as GH deficiency, hypothyroidism, and malabsorption) should be considered and excluded. Upon suspicion of CS, co confirmatory laboratory and imaging tests are necessary. Guidelines for the diagnosis of pediatric CS have been published [12]: the first step is to confirm or rule out the presence of CS and secondly to determine the etiology. A reasonable approach to confirm the inappropriate cortisol secretion is based on urinary free cortisol, midnight serum or salivary cortisol levels, and serum cortisol levels after low dose dexamethasone suppression test. Elevated urinary free cortisol, better if corrected for body surface area [12], should be confirmed on three consecutive 24-hour urinary collections. This test represents the first line investigation and has high sensitivity but low specificity. Midnight serum cortisol greater than 18 ng/mL is considered by some authors [5], the best discriminator in the diagnosis of CS, even if in some cases cortisol nadir is reached earlier than midnight. Late salivary cortisol measurement can have logistical benefits in children [13]. A dexamethasone dosage of 0.5 mg 6 hourly is generally used to perform low dose dexamethasone suppression test in children weighing ≥40 kg, while, in patients <40 kg, a dosage of 30 mcg/kg/day is recommended by guidelines [12]. A serum cortisol below 50 nmol/L after suppression test is considered normal.

**Figure 1:**
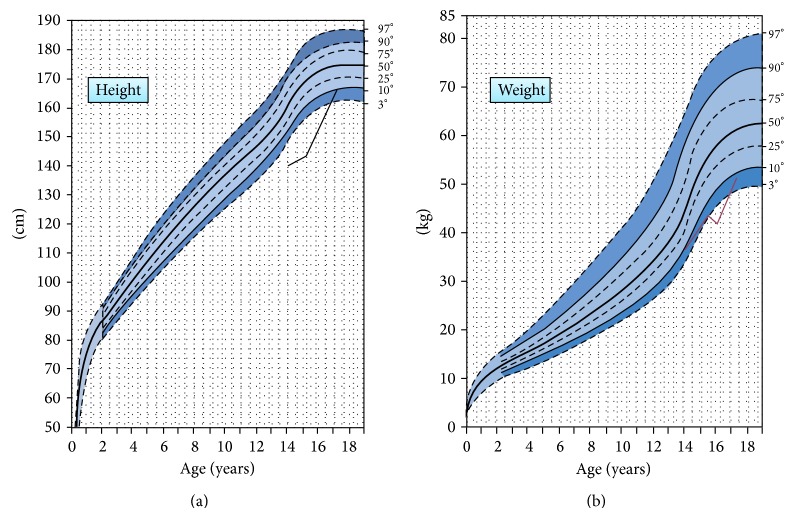
After the remission of hypercortisolism, a rapid catch-up growth was documented during three years of follow-up. Body mass index normalized and pubertal development was adequate.

In our patient first line tests confirmed an inappropriate cortisol secretion, and the detectable ACTH levels were suggestive for ACTH-dependent CS. While in adult patient a “detectable” ACTH value (>2.22 pmol/L) is suggestive of an ACTH-dependent CS, in children a cut-off value of 6.44 pmol/L has been reported to have a sensitivity of 70% in identifying an ACTH-dependent form of CS [14]. In our case an ACTH value of 21.7 pmol/L was clearly suggestive of an ACTH-dependent CS, thus leading to perform the high-dose dexamethasone suppression test to exclude an ectopic ACTH secretion. This test, performed by administering 2 mg of dexamethasone every 6 hours over a 48-hour period, showed an adequate serum cortisol suppression, hence excluding the clinical suspicion of ectopic ACTH syndrome, which indeed is an extremely rare condition among the pediatric population, in contrast with adults where it accounts for about 15% of ACTH-dependent CS [15]. The vast majority of pediatric CD is caused by ACTH-producing pituitary microadenomas, while macroadenomas are extremely rare [16]. Localization of CD by pituitary MRI represents an important tool for the clinicians and should be performed with high resolution method and always with gadolinium to allow detection of microadenomas (only macroadenoma will be detected without contrast). Following administration of gadolinium, more than 90% of ACTH-secreting tumors appear as hypoenhanced lesions. It should be noted that classic spin echo MRI is able to detect only approximately 50% of pituitary lesions [1]. A greater sensitivity seems to be obtained using a post-contrast spoiled gradient-recalled MRI [17]. In our patient the pituitary MRI showed both left and right lesions of 9 mm and 6 mm, respectively: although the radiological finding of the left lesion was suggestive of an ACTH-secreting adenoma, we still preferred to perform bilateral inferior petrosal sinus sampling (BIPSS) [18]: according to the methodology described in the literature, we measured ACTH at baseline and at 3, 5, and 10 minutes after CRH administration. Patients with an ACTH-secreting pituitary adenoma have at least a 2-to-1 central-to-peripheral gradient at baseline or a 3-to-1 central-to-peripheral gradient after stimulation with CRH. In our patient, there were a clear central-to-peripheral gradient and a left-to-right gradient, confirming the suspicion of a left ACTH-secreting microadenoma, with a concomitant right pituitary “incidentaloma,” which can occur in 6% of pediatric population [19]. Some authors report limitations of BPISS in determining the lateralization of ACTH secreting adenoma in pediatric CD, stating that the integration of MRI findings and BPISS cannot predict the location of the tumor more frequently than MRI alone [20]. Moreover, false negative sampling results can occur in presence of a unilateral hypoplastic or plexiform inferior petrosal sinus which results in anomalous venous drainage from the pituitary gland [21]. In our case the patient underwent successfully transsphenoidal surgery which represents the treatment of choice in pediatric CD and which has a similar remission rate as in adults [22]. The pathology report confirmed the diagnosis of pituitary ACTH secreting tumor, and the success of the surgery was established by the reduction in ACTH and serum cortisol levels in the postoperative period and the patient's requirement for hydrocortisone replacement therapy. The patient's pituitary function was periodically monitored to exclude deficiencies of other pituitary hormones. Indeed, during the postsurgical period, a GH hyposecretion induced by supraphysiological glucocorticoid secretion can be observed for at least one year after surgery, with a return to normal GH secretion in about 18 months after successful treatment. There is no agreement about the auxological outcome of children successfully cured: in fact, in the majority of studies the catch-up growth is not achieved even after an adequate treatment, and the final height remains compromised [23], underscoring the significance of early detection and treatment. On the other hand, other authors have reported that a long-term catch-up growth and a satisfactory final height can be obtained in these patients [24]. In our patient, a rapid catch-up growth was documented after about 3 years of follow-up, with a normalization of body mass index and an adequate pubertal development (Figure 1).

## 4. Conclusions

We described a rare case of pediatric Cushing's disease, characterized by the association between ACTH-secreting pituitary adenoma and pituitary incidentaloma: one of the most reliable indicators of hypercortisolism in these patients is growth failure associated with weight gain. Laboratory data and pituitary MRI are very important tools to confirm the clinical suspicion. In our case, BIPSS was necessary to lateralize the site of ACTH production, because of the coexistence of an ACTH-secreting microadenoma and a pituitary “incidentaloma.” Transsphenoidal surgery led to a successful remission of the hypercortisolism, followed by a dramatic improvement in the patient's auxological parameters.

## Figures and Tables

**Table 1:** Bilateral inferior petrosal sinus sampling after CRH stimulation.

	0′	5′	10′	15′	30′	60′
Serum cortisol (nmol/L) (brachial vein)	910	1021	1132	1230	1297	1109
ACTH (pmol/L) (brachial vein)	23	33	55	67	66	53
ACTH (pmol/L) (right petrosal sinus)	22	19	20	24	27	23
ACTH (pmol/L) (left petrosal sinus)	58	124	203	253	220	201

## References

[1] Magiakou M. A., Mastorakos G., Oldfield E. H., Gomez M. T., Doppman J. L., Cutler G. B., Nieman L. K., Chrousos G. P. (1994). Cushing's syndrome in children and adolescents: presentation, diagnosis, and therapy. *The New England Journal of Medicine*.

[2] Hayles A. B., Hahn H. B., Sprague R. G., Bahn R. C., Priestley J. T. (1966). Hormone-secreting tumors of the adrenal cortex in children. *Pediatrics*.

[3] Magiakou M. A., Mastorakos G., Gomez M. T., Rose S. R., Chrousos G. P. (1994). Suppressed spontaneous and stimulated growth hormone secretion in patients with Cushing's disease before and after surgical cure. *The Journal of Clinical Endocrinology and Metabolism*.

[4] Stratakis C. A. (2012). Cushing syndrome in pediatrics. *Endocrinology & Metabolism Clinics of North America*.

[5] Chan L. F., Storr H. L., Grossman A. B., Savage M. O. (2007). Pediatric Cushing's syndrome: clinical features, diagnosis, and treatment. *Arquivos Brasileiros de Endocrinologia e Metabologia*.

[6] Weber A., Trainer P. J., Grossman A. B., Afshar F., Medbak S., Perry L. A., Plowman P. N., Rees L. H., Besser G. M., Savage M. O. (1995). Investigation, management and therapeutic outcome in 12 cases of childhood and adolescent Cushing's syndrome. *Clinical Endocrinology*.

[7] Lee P. D. K., Winter R. J., Green O. C. (1985). Virilizing adrenocortical tumors in childhood: eight cases and a review of the literature. *Pediatrics*.

[8] Savage M. O., Lienhardt A., Lebrethon M.-C., Johnston L. B., Huebner A., Grossman A. B., Afshar F., Plowman P. N., Besser G. M. (2001). Cushing's disease in childhood: presentation, investigation, treatment and long-term outcome. *Hormone Research*.

[9] Leong G. M., Abad V., Charmandari E. (2007). Effects of child- and adolescent-onset endogenous Cushing syndrome on bone mass, body composition, and growth: a 7-year prospective study into young adulthood. *Journal of Bone and Mineral Research*.

[10] Briassoulis G., Damjanovic S., Xekouki P., Lefebvre H., Stratakis C. (2011). The glucocorticoid receptor and its expression in the anterior pituitary and the adrenal cortex: a source of variation in hypothalamic-pituitary-adrenal axis function; Implications for pituitary and andrenal tumors. *Endocrine Practice*.

[11] Briassoulis G., Horvath A., Christoforou P., Lodish M., Xekouki P., Quezado M., Patronas N., Keil M. F., Stratakis C. A. (2012). Lack of mutations in the gene coding for the hGR ( NR3C1 ) in a pediatric patient with ACTH-secreting pituitary adenoma, absence of stigmata of Cushing's syndrome and unusual histologic features. *Journal of Pediatric Endocrinology and Metabolism*.

[12] Magiakou M. A., Chrousos G. P. (2002). Cushing's syndrome in children and adolescents: current diagnostic and therapeutic strategies. *The Journal of Endocrinological Investigation*.

[13] Gafni R. I., Papanicolaou D. A., Nieman L. K. (2000). Nighttime salivary cortisol measurement as a simple, noninvasive, outpatient screening test for Cushing's syndrome in children and adolescents. *Journal of Pediatrics*.

[14] Batista D. L., Riar J., Keil M., Stratakis C. A. (2007). Diagnostic tests for children who are referred for the investigation of Cushing syndrome. *Pediatrics*.

[15] Isidori A. M., Kaltsas G. A., Pozza C. (2006). Extensive clinical experience—the ectopic adrenocorticotropin syndrome: Clinical features, diagnosis, management, and long-term follow-up. *The Journal of Clinical Endocrinology & Metabolism*.

[16] Stratakis C. A., Schussheim D. H., Freedman S. M., Keil M. F., Pack S. D., Agarwal S. K., Skarulis M. C., Weil R. J., Lubensky I. A., Zhuang Z., Oldfield E. H., Marx S. J. (2000). Pituitary macroadenoma in a 5-year-old: an early expression of multiple endocrine neoplasia type 1. *Journal of Clinical Endocrinology and Metabolism*.

[17] Batista D., Courkoutsakis N. A., Oldfield E. H., Griffin K. J., Keil M., Patronas N. J., Stratakis C. A. (2005). Detection of adrenocorticotropin-secreting pituitary adenomas by magnetic resonance imaging in children and adolescents with cushing disease. *Journal of Clinical Endocrinology and Metabolism*.

[18] Storr H. L., Afshar F., Matson M., Sabin I., Davies K. M., Evanson J., Plowman P. N., Besser G. M., Monson J. P., Grossman A. B., Savage M. O. (2005). Factors influencing cure by transsphenoidal selective adenomectomy in paediatric Cushing's disease. *European Journal of Endocrinology*.

[19] Tamura T., Tanaka R., Korii K., Okazaki H. (2000). Pediatric pituitary adenoma. *Endocrine Journal*.

[20] Batista D., Gennari M., Riar J., Chang R., Keil M. F., Oldfield E. H., Stratakis C. A. (2006). An assessment of petrosal sinus sampling for localization of pituitary microadenomas in children with cushing disease. *Journal of Clinical Endocrinology and Metabolism*.

[21] Doppman J. L., Chang R., Oldfield E. H., Chrousos G., Stratakis C. A., Nieman L. K. (1999). The hypoplastic inferior petrosal sinus: a potential source of false- negative results in petrosal sampling for Cushing's disease. *Journal of Clinical Endocrinology and Metabolism*.

[22] Lienhardt A., Grossman A. B., Dacie J. E. (2001). Relative contributions of inferior petrosal sinus sampling and pituitary imaging in the investigation of children and adolescents with ACTH-dependent Cushing's syndrome. *The Journal of Clinical Endocrinology & Metabolism*.

[23] Magiakou M. A., Mastorakos G., Chrousos G. P. (1994). Final stature in patients with endogenous Cushing's syndrome. *Journal of Clinical Endocrinology and Metabolism*.

[24] Lebrethon M.-C., Grossman A. B., Afshar F., Plowman P. N., Besser G. M., Savage M. O. (2000). Linear growth and final height after treatment for Cushing's disease in childhood. *Journal of Clinical Endocrinology and Metabolism*.

